# Potential Molecular Mechanisms of Plantain in the Treatment of Gout and Hyperuricemia Based on Network Pharmacology

**DOI:** 10.1155/2020/3023127

**Published:** 2020-10-22

**Authors:** Pei Liu, Huachong Xu, Yucong Shi, Li Deng, Xiaoyin Chen

**Affiliations:** College of Traditional Chinese Medicine, Jinan University, Guangzhou 510632, China

## Abstract

**Background:**

The incidence of gout and hyperuricemia is increasing year by year in the world. Plantain is a traditional natural medicine commonly used in the treatment of gout and hyperuricemia, but the molecular mechanism of its active compounds is still unclear. Based on network pharmacology, this article predicts the targets and pathways of effective components of plantain for gout and hyperuricemia and provides effective reference for clinical medication.

**Method:**

Traditional Chinese medicine systems pharmacology database and analysis platform (TCMSP) and SymMap databases were used to screen out the active compounds and their targets in plantain. GeneCards, Therapeutic Target Database (TTD), and Online Mendelian Inheritance in Man (OMIM) databases were used to find the targets corresponding to gout and hyperuricemia. Venn diagram was used to obtain the intersection targets of plantain and diseases. The interaction network of the plantain active compounds-targets-pathways-diseases was constructed by using Cytoscape 3.7.2 software. Finally, Gene Ontology (GO) and Kyoto Encyclopedia of Genes and Genomes (KEGG) enrichment analyses were carried out.

**Result:**

Seven active compounds were identified by network pharmacological analysis, including dinatin, baicalein, baicalin, sitosterol, 6-OH-luteolin, stigmasterol, and luteolin. Plantain plays a role in gout and hyperuricemia diseases by regulating various biological processes, cellular components, and molecular functions. The core targets of plantain for treating gout are MAPK1, RELA, TNF, NFKBIA, and IFNG, and the key pathways are pathways in cancer, hypoxia-inducible factor-1 (HIF-1) signaling pathway, interleukin (IL)-17 signaling pathway, Chagas disease (American trypanosomiasis), and relaxin signaling pathway. The core targets of plantain for hyperuricemia are RELA, MAPK1, NFKBIA, CASP3, CASP8, and TNF, and the main pathways are pathways in cancer, apoptosis, hepatitis B, IL-17 signaling pathway, and toxoplasmosis.

**Conclusion:**

This study explored the related targets and mechanisms of plantain for the treatment of gout and hyperuricemia from the perspective of network pharmacological analysis, reflecting the characteristics of multiple components, multiple targets, and multiple pathways, and it provides a good theoretical basis for the clinical application of plantain.

## 1. Introduction

Gout and hyperuricemia are common clinical diseases. Hyperuricemia is the basis of gout disease, manifested by an increase in serum uric acid (UA) content (UA ≥ 360 *μ*mol/L). When the serum UA level rises above the normal threshold, monosodium urate (MSU) crystals are deposited on the joints, causing joint damage and inflammatory reaction and finally suffering from gout [1, 2]. The pathological threshold for hyperuricemia is defined as 6.8 mg/dl [3]. The gold standard of gout diagnosis is to detect the presence of MSU crystals in synovial fluid of joint with polarized light microscope [3]. Incidence and prevalence of gout and hyperuricemia are increasing [4]. The global prevalence of gout is between 0.1% and 10%; the prevalence of hyperuricemia in the United States is 21.2% for men and 21.6% for women [5]. Therefore, reducing the content of serum UA is the main measure to prevent and treat gout and hyperuricemia. If not controlled, it will lead to a high incidence, reduce the quality of life, and even cause disability. At present, there are different degrees of adverse reactions to UA-lowering drugs, such as allopurinol, febuxostat, or benzbromarone [6]. Febuxostat and allopurinol have a higher incidence of acute renal failure than other drugs [7], and allopurinol and benzbromarone cause different degrees of acute liver injury [8]. Therefore, it is urgent to find more natural drugs with less number of side effects for the treatment of gout and hyperuricemia.

Plantain is a natural medicine with heat-clearing-dampness-removing, detoxification, and antiphlogistic effect. It plays a role in the treatment of ulcer, diabetes, diarrhea, inflammation, viral infections, etc. [9]. In the traditional treatment, plantain has been used in the clinical treatment of gout and hyperuricemia with remarkable effect. Modern studies have also shown that plantain has the effect of reducing serum UA [10, 11], anti-inflammatory and analgesic [12, 13], inhibiting xanthine oxidase (XOD) activity [14], and protecting the kidney [15]. However, the mechanism of plantain to treat gout and hyperuricemia is still unclear, especially the molecular target mechanism of its effective chemical components, such as specific targets and pathways, which needs to be further explored.

As a new research method, network pharmacology has been widely used in the field of traditional Chinese medicine (TCM). Some studies use the network pharmacology method to use the existing database information to initially explore the target and pathway relationships between drugs and diseases and on this basis to clarify the molecular mechanism. This shows that network pharmacology is a relatively efficient and easy-to-implement method for studying the molecular mechanism of TCM. Therefore, this article used the method of network pharmacological analysis [16] to explore the target molecules of plantain which acts on patients with gout and hyperuricemia and established the network of plantain active compounds-targets-pathways-diseases, to provide new ideas for clinical treatment and drug research.

## 2. Materials and Methods

### 2.1. Screening of Plantain Active Compounds

Using “cheqiancao” as a key word, the components of plantain are searched in TCMSP (http://tcmspw.com/tcmsp.php), a pharmacological database and analysis platform of Chinese medicine system. It contains 499 Chinese herbal medicines registered in the Chinese Pharmacopoeia, containing a total of 29384 chemicals, 837 related diseases, and 3311 targets [17], which provides powerful assistance for studying the mechanism of action of Chinese herbal medicines. Bioavailability (OB) and drug-likeness (DL) are important indexes to evaluate ADME (absorption, distribution, metabolism, and excretion) properties and are key factors to screen active compounds of drugs [17]. In order to search the active compounds of plantain more comprehensively, this paper selected them on the condition of OB ≥ 30% and DL ≥ 0.18.

### 2.2. Collection of Active Compound Targets of Plantain

Take “Cheqiancao” as the key word and input it into the SymMap (https://www.symmap.org) and the TCMSP databases to find the targets corresponding to its active compounds. SymMap database is a comprehensive database of TCM with enhanced symptom map. It contains 1717 TCM symptoms, 961 modern medicine symptoms, 499 herbs, 19595 ingredients, 4302 targets, and 5235 diseases [18], which are related to each other.

### 2.3. Collection of Disease Targets and Screening of Intersection Targets

With the keywords of “gout”, “gout arthritis”, “gouty arthritis”, and “hyperuricemia”, target genes related to gout and hyperuricemia were found in GeneCards (https://www.genecards.org), TTD (http://db.idrblab.net/ttd/), and OMIM (https://www.omim.org) databases. The GeneCards database includes more than 7000 human genes, and each gene has an approved gene symbol [19]. TTD database contains 2360 targets and 20600 drugs [20]. The OMIM database is a knowledge base of human genes and hereditary diseases. As of September 13, 2004, OMIM contains 10208 entries describing genes with known sequences and 5777 entries describing phenotypes [21]. The three methods have a good reference for the collection of disease targets. According to the targets of plantain and disease, the repeated targets of the two were screened by Excel, and their intersection targets were obtained. According to their intersection targets, get the Venn diagram in the website https://bioinfogp.cnb.csic.es/tools/venny/.

### 2.4. Construction of Protein-Protein Interaction Network

High-throughput yeast protein-protein interaction (PPI) datasets include Gavin dataset, Krogan dataset, Munich Information Center for Protein Sequences (MIPS) dataset, and STRING dataset. The STRING dataset is now one of the largest PPI datasets, including coexpression data, biomedical literature data, high-throughput data, and genomic background data [22]. Therefore, the intersection targets of plantain and gout and plantain and hyperuricemia were input into string (https://string-db.org) [23] to construct the protein-protein interaction network. In order to ensure the high confidence of information, the scoring condition was set to >0.90, and the isolated proteins in the figure were hidden. The selected target proteins were limited to “*Homo sapiens*.”

### 2.5. Construction of the Network Model

The active compounds of plantain and their corresponding targets and the intersection targets of diseases and plantain were sorted out and input into Cytoscape 3.7.2 to construct the following networks: (1) network between plantain active compounds and their corresponding targets; (2) network between plantain active compounds, intersection targets, and diseases. Cytoscape is a kind of software which can express the interaction between protein and protein, protein and DNA or gene efficiently and can visualize network relationships [24].

### 2.6. GO Enrichment Analysis and KEGG Pathway Enrichment Analysis

In order to further explore the pathways of the disease, the intersection targets of plantain and disease were annotated, and gene ID conversion was carried out in DAVID Bioinformatics Resources 6.7 (https://david-d.ncifcrf.gov). DAVID Bioinformatics Resources is a comprehensive biological knowledge base and analysis tool, which is mainly analyzed by gene function classification, function annotation graph or clustering, and function annotation table [25]. Then, the intersection targets were input into OmicShare (https://www.omicshare.com/) [26] in ensemble gene ID format for GO and KEGG enrichment analysis. GO enrichment analysis included molecular function analysis, cell component analysis and biological process analysis [27], and the KEGG analysis [28] and compared the top 20 pathways.

### 2.7. Plantain Active Compounds-Targets-Pathways Analysis

The top 15 pathways obtained from the above KEGG pathway enrichment analysis correspond to the intersection targets and active compounds of plantain in the treatment of gout and hyperuricemia, respectively, and construct the network of “plantain active compounds-targets-signaling pathways.”

## 3. Results

### 3.1. Screening of Plantain Active Compounds

With OB ≥ 30% and DL ≥ 0.18 as the screening conditions, a total of 10 potential active compounds satisfying the conditions are obtained, as shown in Table 1. The 10 potential active compounds of plantain were input into SymMap and TCMSP databases to find the corresponding targets. The results show that only 7 compounds found the corresponding targets, respectively: dinatin, baicalein, baicalin, sitosterol, 6-OH-luteolin, stigmasterol, and luteolin; they are the main active compounds of plantain in the treatment of gout and hyperuricemia. Among them, dinatin, baicalein, baicalin, 6-OH-luteolin, and luteolin are flavonoids in plantain, while sitosterol and stigmasterol are triterpenes and steroids. It can be seen that the compounds for treating gout and hyperuricemia are mainly concentrated in flavonoids, triterpenes, and steroids. The chemical abstracts service number, chemical structure, molecular formula, and molecular weight are shown in Table 2. In the treatment of rheumatoid arthritis (RA) and osteoarthritis (OA), flavonoids and triterpenoids have been shown to have good anti-inflammatory effects by inhibiting matrix metalloproteinase (MMP) and cyclooxygenase-2 (COX-2), reducing the production of inflammatory cytokines and chemokines such as tumor necrosis factor-*α* (TNF-*α*), IL-1, IL-6, and chemokine C-C motif ligand 5 (CCL5) by acting on different targets in the NF-*κ*B (nuclear factor-kappa B) signaling pathway [29]. Flavonoids also can inhibit the levels of IL-1*β*, IL-2, IL-6, TNF-*α*, and IL-17A, block the NF-*κ*B signaling pathway and nucleotide binding oligomerization domain-like receptor protein 3 (NLRP3) inflammatory factors, and enhance the immune response to achieve anti-inflammatory purposes [30]. At present, there are few studies on the active compounds and mechanism of plantain in the treatment of gout and hyperuricemia, which need to be further researched.

### 3.2. Collection of Plantain Active Compounds and Network Construction

**Figure 1:**
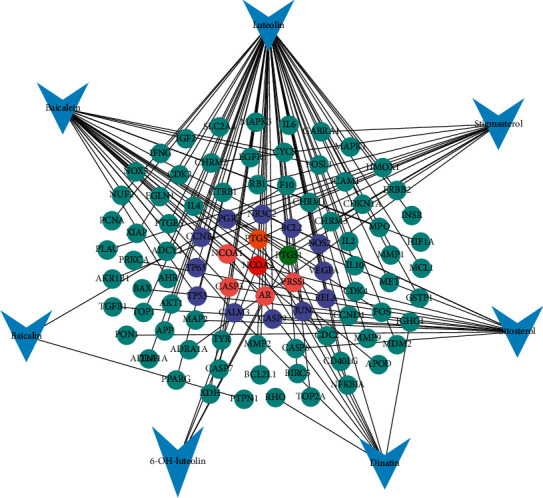
Plantain active compounds-targets network diagram. Note: the blue arrow nodes represent the active compounds of plantain and the circular nodes represent the active compounds targets (blue means one active compound corresponds to this target, purple means two active compounds correspond to this target, pink means three active compounds correspond to this target, orange means four active compounds correspond to this target, green means five active compounds correspond to this target, and red means 6 active compounds correspond to this target). Edges represent the interactions between compounds and the targets.

Seven active compounds of plantain were input into SymMap and TCMSP databases one by one, and the total number of corresponding targets was 92. The active compounds and targets are imported into Cytoscape 3.7.2 software for network construction. As shown in Figure 1, the network between plantain active compounds and corresponding targets consists of 99 nodes (7 active compound nodes and 92 corresponding target nodes) and 124 edges. The active compound luteolin has the most number of targets. It can be seen from the figure that one active compound can correspond to multiple targets, and one target can correspond to multiple active compounds; the more towards the center, the more active compounds the target corresponds to. It reflects the characteristics of multicomponent and multitarget of plantain.

### 3.3. Collection of Disease Targets Information and Screening of Intersection Targets

**Figure 2:**
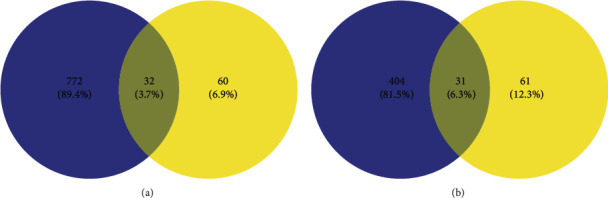
(a) Intersection targets of plantain and gout. (b) Intersection targets of plantain and hyperuricemia. Note: blue represents the number of targets for gout or hyperuricemia, yellow represents the number of targets for active constituents of plantain, and the middle part represents the intersection targets of the two.

We found 804 targets corresponding to gout and 435 targets corresponding to hyperuricemia in GeneCards, TTD, and OMIM databases. Based on plantain and disease targets, get the Wayne diagrams in https://bioinfogp.cnb.csic.es/tools/venny/. As shown in Figures 2(a) and 2(b), there were 32 intersection targets of plantain and gout and 31 intersection targets with hyperuricemia.

### 3.4. Construction of Protein Interaction Network

**Figure 3:**
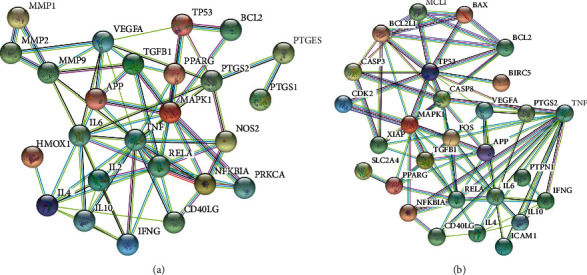
(a) Protein interaction network of plantain for gout. (b) Protein interaction network of plantain for hyperuricemia. Note: the circular nodes represent the interacting proteins that directly or indirectly interact with each other. The structures in the nodes are the protein structures, and the edges represent protein-to-protein interactions.

Figures 3(a) and 3(b) are protein interaction network diagrams of plantain for gout and hyperuricemia, respectively. The nodes in the figure represent proteins, and the structure in the nodes is the protein structure. It can be seen from the figure that there is a correlation between the targets, which indicates that plantain may have a therapeutic effect on gout and hyperuricemia through the coordination of these targets in multiple pathways and ways.

### 3.5. Plantain Active Compounds-Intersection Targets-Disease Network

**Figure 4:**
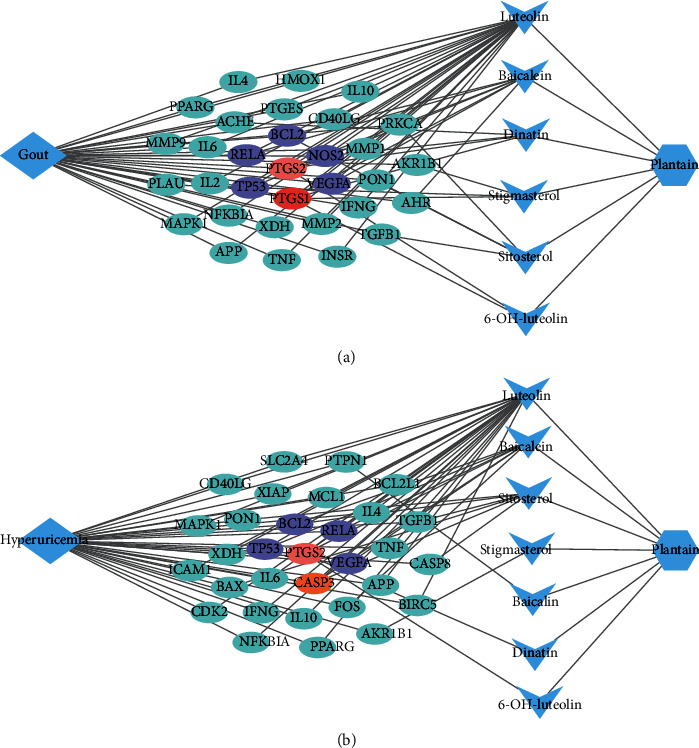
(a) Plantain active compounds-intersection targets-gout correlation network. (b) Plantain active compounds-intersection targets-hyperuricemia correlation network. Note: the blue diamond nodes represent disease, the blue hexagon nodes represent plantain, the blue triangles represent the active compounds of the drug, and the circular nodes represent the intersection targets (blue means only one active compound corresponding to this target, purple means two active compounds acting on this target, orange means there are 3 active compounds corresponding to this target, pink means there are 4 active compounds acting on this target, and red means 5 active compounds acting on this target). The edges represent the interactions between the nodes.

As shown in Figures 4(a) and 4(b), they are Cytoscape network diagrams of plantain active compounds-targets-gout and plantain active compounds-targets-hyperuricemia, respectively. The gout network diagram consists of 40 nodes (32 target nodes, 6 active compound nodes, 1 gout disease node, and 1 plantain node) and 82 edges. The hyperuricemia network diagram consists of 40 nodes (31 target nodes, 7 active compound nodes, 1 hyperuricemia disease node, and 1 plantain node) and 78 edges. The more towards the center, the more active compounds the target corresponds to. These targets connect the relationship between the active compounds of plantain and disease and provide a better reference for exploring the mechanism of plantain in the treatment of gout and hyperuricemia.

### 3.6. GO Enrichment Analysis and KEGG Pathway Enrichment Analysis

**Figure 5:**
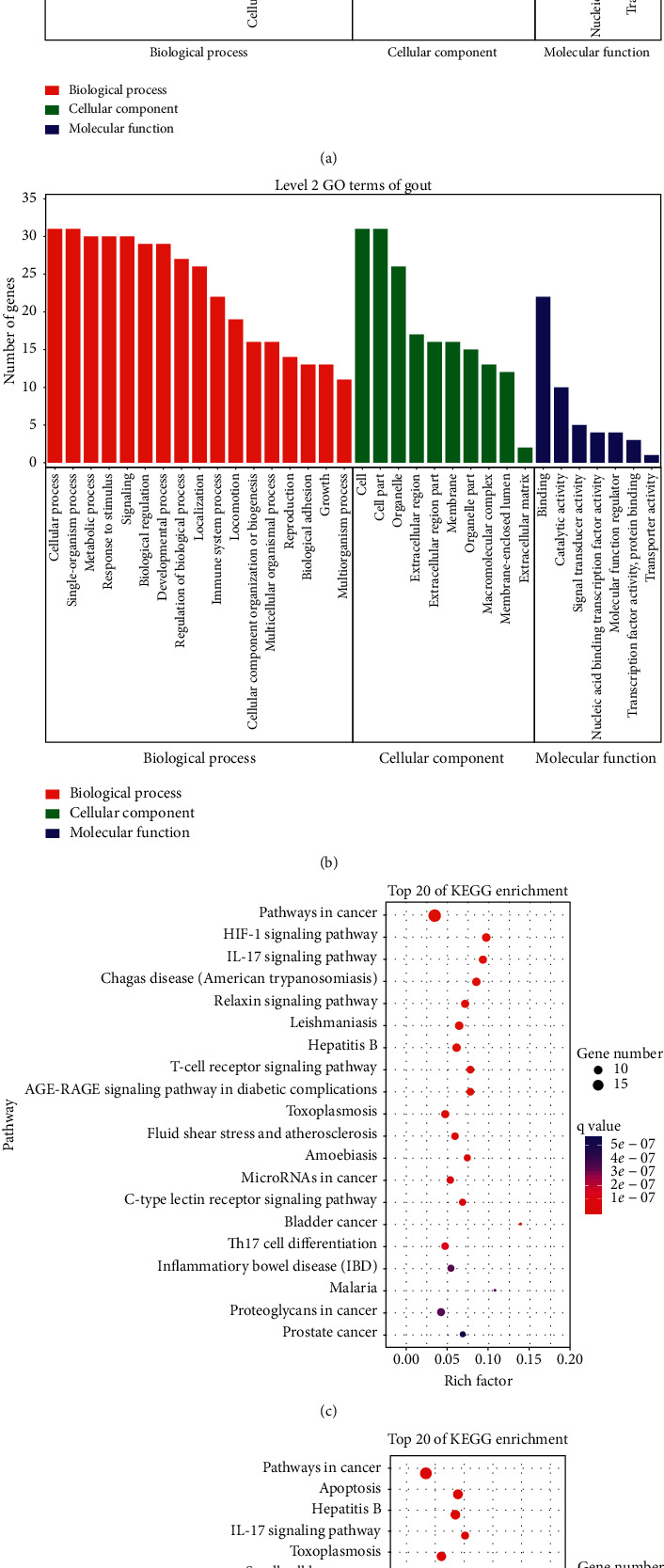
(a) GO enrichment analysis of gout disease. (b) GO enrichment analysis of hyperuricemia disease. Note: red represents biological processes, green represents cellular component, and blue represents molecular function. (c) KEGG enrichment analysis of gout disease. (d) KEGG enrichment analysis of hyperuricemia disease. Note: the *x*-axis represents the gene ratio, the *y*-axis represents the enriched pathways; the size of the dots indicates the gene number; the color of the dots represents the level of *P* value.

In order to study the mechanism of plantain on gout and hyperuricemia more systematically, OmicShare was used for GO and KEGG enrichment analysis. Figures 5(a) and 5(b) are the results of GO enrichment analysis of plantain in the treatment of gout and hyperuricemia. The results showed that plantain acts on gout by regulating several biological processes (Figure 5(a)), among which the top five are cellular process, single-organism process, metabolic process, response to stimulus, and signaling. The top five cellular components are cell, cell part, organelle, extractable region, and extractable region part. At the molecular function level, plantain mainly regulates binding and catalytic activity on gout. Plantain acts on hyperuricemia through a variety of biological processes (Figure 5(b)), the top five are cellular process, single-organism process, biological regulation, metabolic process and response to stimulus. The top five cellular components are cell, cell part, organelle, macromolecular complex, and extracellular region. At the molecular level, plantain also mainly regulates binding and catalytic activity on hyperuricemia.

Figures 5(c) and 5(d) show the bubble chart results of KEGG enrichment analysis of plantain in the treatment of gout and hyperuricemia. Only the top 20 pathways were compared and analyzed. The data show that plantain mainly regulates pathways in cancer, HIF-1 signaling pathway, IL-17 signaling pathway, Chagas disease (American trypanosomiasis), and relaxin signaling pathway to treat gout (Figure 5(c)) and mainly regulates pathways in cancer, apoptosis, hepatitis B, IL-17 signaling pathway, and toxoplasmosis (Figure 5(d)) to treat hyperuricemia.

### 3.7. Plantain Active Compounds-Intersection Targets-Signaling Pathways Network

**Figure 6:**
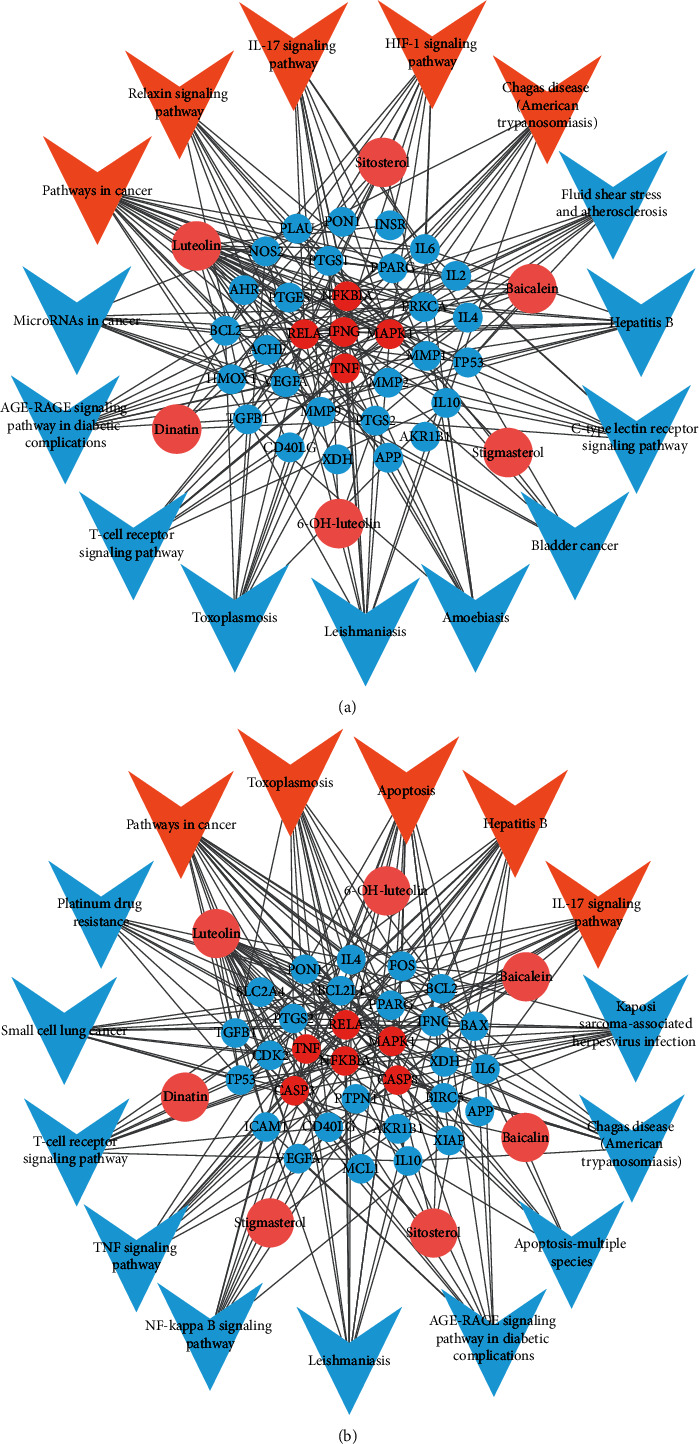
(a) Active compounds-intersection targets-signaling pathways network of plantain in the treatment of gout. (b) Active compounds-intersection targets-signaling pathways network of plantain in the treatment of hyperuricemia. Note: the pink circular nodes represent the active compounds in plantain, the remaining circular nodes represent the targets, among which the red circular nodes represent the key targets; the arrow nodes represent the regulation pathways, among which the orange nodes are the important pathways; the edges represent interaction among the three.

In order to further explore the relationship between compounds, intersection targets, and signaling pathways, the top 15 pathways obtained by KEGG enrichment analysis correspond to the targets and components of plantain in the treatment of gout and hyperuricemia, respectively, and construct a network diagram of active compounds-intersection targets-signaling pathways. The results are shown in Figures 6(a) and 6(b). The signaling pathway figure of gout disease consists of 53 nodes (15 signaling pathway nodes, 6 active compound nodes, and 32 intersection target nodes) and 191 edges. The signaling pathway figure of hyperuricemia disease is composed of 53 nodes (15 signaling pathway nodes, 7 active compound nodes, and 31 intersection target nodes) and 208 edges. Each pathway corresponds to multiple targets, and each target is connected to multiple pathways, reflecting the multicomponent, multitarget, multipathway mechanism of plantain treatment of gout and hyperuricemia. Multiple pathways are linked to each other by common targets, indicating that each pathway plays a synergistic role in treating gout and hyperuricemia diseases.

## 4. Discussion

### 4.1. Summary of the Mechanism of Plantain in the Treatment of Gout and Hyperuricemia

Gout is a chronic disease in which urate crystals are deposited in the joints and cause inflammation; the prevalence rates of men and women are 5.9% and 2.0%, respectively [2]. Hyperuricemia is the basis of gout. The prevalence rates of men and women are 21.2% and 21.6%, respectively [2]. At present, there is no perfect treatment for gout and hyperuricemia. Western medicine is the main treatment, but long-term use will cause side effects. Based on the network pharmacological analysis, this study explored the active compounds and mechanism of plantain in the treatment of gout and hyperuricemia. Seven active compounds were obtained from TCMSP database: dinatin, baicalein, baicalin, sitosterol, 6-OH-luteolin, stigmasterol, and luteolin. There are 32 intersection targets of gout and active compounds of plantain; the key targets are MAPK1, RELA, TNF, NFKBIA, and IFNG and the key pathways include pathways in cancer, HIF-1 signaling pathway, IL-17 signaling pathway, Chagas disease (American trypanosomiasis), and relaxin signaling pathway. There are 31 intersection targets for hyperuricemia, the main targets are RELA, MAPK1, NFKBIA, CASP3, CASP8, and TNF, and the main pathways include pathways in cancer, apoptosis, hepatitis B, IL-17 signaling pathway, and toxoplasmosis. In addition, plantain also regulates 17 biological processes, 10 cell components, and 7 molecular functions to treat gout and hyperuricemia. This indicates that plantain is composed of a variety of compounds and acts on different targets of gout and hyperuricemia through multiple pathways, which fully reflects the characteristics of multiple components, multiple targets, and multiple pathways, and provides the basis for clinical medication and scientific verification in the later stage.

According to the results of KEGG enrichment analysis, the following pathways related to the treatment of gout and hyperuricemia were selected for analysis: IL-17 signaling pathway, HIF-1 signaling pathway, relaxin signaling pathway, TNF signaling pathway, and advanced glycation end products-receptor for advanced glycation end products (AGE-RAGE) signaling pathway in diabetic complications.

### 4.2. IL-17 Signaling Pathway

**Figure 7:**
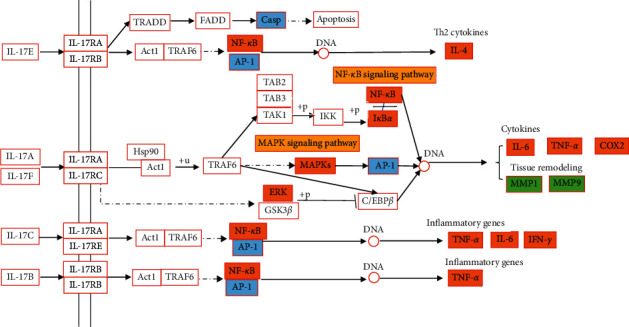
IL-17 signaling pathway of plantain in the treatment of gout and hyperuricemia. Note: orange is the common target of gout and hyperuricemia, green is the target of gout, blue is the target of hyperuricemia, and yellow is the other signaling pathways that may be involved in this pathway.

In the early stages of acute gouty arthritis, local inflammation is strengthened, with joint redness and severe pain [31]. Among them, IL-17 is an important proinflammatory factor, which plays an important role in inflammation and immune response [32].  Figure 7 shows the results of IL-17 signaling pathway analysis. Experimental studies have shown that IL-17 combines with its receptor and activates downstream pathways including NF-*κ*B and MAPK, thus inducing the expression of proinflammatory cytokines such as IL-6 and TNF-*α* to induce inflammation [32, 33]. Intra-articular injection of IL-17 can increase the production of TNF-*α*, IL-1*β*, and CXCL1/KC (chemokine (C-X-C motif) ligand 1/keratinocyte-derived chemokine), mediate the recruitment of neutrophils, and induce hyperalgesia and arthritis [34]. In patients with RA, T and B lymphocytes can stimulate the production of proinflammatory mediators such as IL-17, TNF-*α*, IL-1, and IL-6 [35]. IL-17 mediates the activation of NF-*κ*B pathway by activating related receptors; meanwhile, inflammatory cytokines such as TNF-*α* and IL-1*β* can also enhance the activity of IL-17 and promote the production of matrix metalloproteinase MMP1/3/9/13 [32]. In patients with gouty arthritis, the NF-*κ*B and activator protein 1 (AP-1) pathways are activated [36], and the expression of proinflammatory cytokines IL-1*β*, IL-8, IL-17, TNF-*α*, and NLRP3 inflammasome increased [37]. It has been proved that plantain has good anti-inflammatory and anti-UA activities. It can inhibit the activation of NF-*κ*B and MAPK, inhibit the phosphorylation of inhibitor of NF-*κ*B (I*κ*B*α*), p65, p38, Jun N-terminal kinases (JNK), and extracellular signal-regulated kinase (ERK), decrease the expression of IL-1*β*, TNF-*α*, and IL-6, and promote the secretion of anti-inflammatory factor IL-10 in mice with acute lung injury [38]. Plantain can reduce serum uric acid level of hyperuricemia mice by inhibiting XOD activity and can improve swelling and activity of gouty arthritis [39–41]. Studies have shown that the compounds baicalein [42], baicalin [35], sitosterol [43], stigmasterol [44], and luteolin [45] can inhibit the level of IL-17. In conclusion, plantain has a good therapeutic effect on gouty arthritis and hyperuricemia; however, the mechanism of plantain in the treatment of gouty arthritis and hyperuricemia disease through IL-17 signaling pathway needs to be further explored.

### 4.3. HIF-1 Signaling Pathway

**Figure 8:**
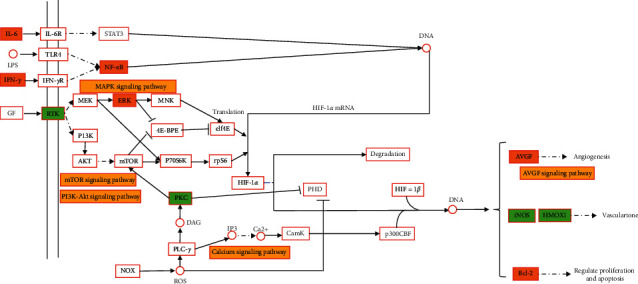
HIF-1 signaling pathway of plantain in the treatment of gout and hyperuricemia. Note: orange is the common target of gout and hyperuricemia, green is the target of gout, and yellow is the other signaling pathways that may be involved in this pathway.

Hypoxia-inducible factor (HIF) is composed of two subunits, HIF-1*α* and HIF-1*β*, and HIF-1*α*-mediated gene transcription regulation has made great progress in cell hypoxia stress [46]. When inflammation occurs, blood flow slows down, and the oxygen consumption of inflammatory cells and antigens increases, resulting in local environmental hypoxia and activation of HIF, which can stably exist and participate in the activation of NF-*κ*B and glycolysis [46]. Glycolysis can trigger inflammatory response, especially plays a key role in the activation of macrophages induced by IL-4, it can also promote the activation of NLRP3 inflammasome and the secretion of inflammatory cytokines such as IL-1*β* and IL-6 [47]. HIF-1*α* can directly or indirectly participate in the regulation of inflammatory factors such as IL-1*β*, NLRP3 [48], so the HIF-1*α* pathway plays a key role in promoting inflammation.  Figure 8 shows the results of HIF-1 signaling pathway analysis. In RA diseases, inflammatory factors mediate the activation of NF-*κ*B pathway [32], TNF-*α* induces the production of vascular endothelial growth factor (VEGF) [49], and it is found that a large amount of HIF-1*α* is expressed in macrophages in the intimal layer and subintimal region of patients [50]. It is inferred that inflammatory cytokines may be important inducers of HIF-1 expression [49]. The expression of HIF-1*α*, nerve growth factor (NGF), hepatocyte growth factor (HGF), and inflammatory cytokines: IL-1*β*, IL-6, TNF-*α*, and chemokines (IL-8 and monocyte chemotactic protein 1 (MCP-1)) were increased in gouty arthritis patients after stimulation with MSU crystals. Meanwhile, the expression of VEGF was activated by NF-*κ*B and AP-1 pathway [36]. Studies have shown that plantain can inhibit the expression of HIF-1*α* and VEGF and the phosphorylation of protein kinase B (Akt) and downregulate the gene expression of TNF-*α* and IL-1*β* and the activation of NF-*κ*B to treat diabetic retinal injury [51]. The compounds dinatin [52], baicalein [53], baicalin [54], and luteolin [55] have been shown to reduce HIF-1 level. Therefore, HIF-1*α* signaling pathway may be the pathway of plantain in the treatment of gouty arthritis, and the specific mechanism and pathway need to be further verified.

### 4.4. Relaxin Signaling Pathway

**Figure 9:**
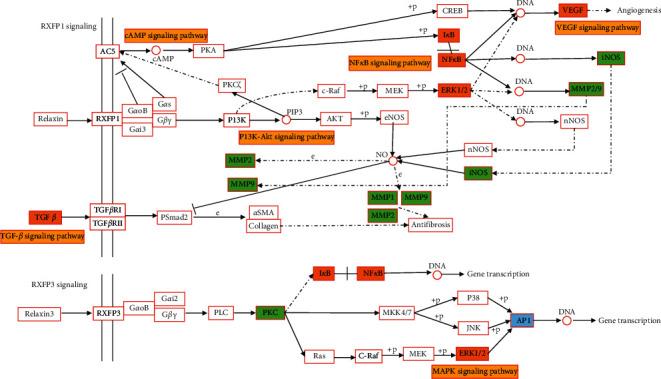
Relaxin signaling pathway in the treatment of gout and hyperuricemia with plantain. Note: orange is the common target of gout and hyperuricemia, green is the target of gout, blue is the target of hyperuricemia, and yellow is the other signaling pathways that may be involved in this pathway.

Relaxin is a polypeptide hormone secreted by the corpus luteum of the ovary. This hormone can promote the healing of skeletal muscles and injured ligaments and can also change the characteristics of cartilage and tendons; it is a regulator of inflammation and fibrosis [56]. During pregnancy, the content of relaxin increases, so a woman's risk of developing arthritis decreased during that period [56].  Figure 9 shows the results of relaxin signaling pathway analysis. In terms of anti-inflammation, relaxin can inhibit the adhesion of neutrophils to endothelial cells and the infiltration of macrophages, can inhibit the activity of NLRP3 inflammasome and NF-*κ*B signaling pathway to reduce the inflammatory reaction, and can reduce the levels of cytokines, such as IL-1*β*, IL-6, and TNF-*α* [57]. For example, relaxin can reduce cardiac inflammatory response by reducing the inflammation mediated by IL-1*β*, IL-6, and NLRP3 [58, 59] and also can inhibit vascular inflammation by inhibiting the expression of TNF-*α* and chemokine C-C motif ligand 2 (CCL2) [60]. Studies have found that relaxin and estrogen can reduce TNF-*α* and vascular endothelial growth factor by increasing the expression of relaxin family peptide receptor (RXFP1) gene and can increase the expression of anti-inflammatory cytokine IL-10 to improve the symptoms of adjuvant-induced RA in rats [61–63]. In terms of antifibrosis effect, relaxin can inhibit Smad2 phosphorylation level and fibrosis mediator transforming growth factor-*β*1(TGF-*β*1) and promote the expression of MMP-1/2/9/13 by activating RXFP1, so as to reduce the production and deposition of collagen to achieve the antifibrosis effect [57]. Previous studies have found that TGF-*β*1 expression was increased in synovium of patients with acute gouty arthritis and in the fibrotic kidney of patients with hyperuricemia [64–66]. It is inferred that relaxin may treat gout and hyperuricemia nephropathy by inhibiting the level of TGF-*β*1. In general, the relaxin signaling pathway plays a positive role in the improvement of arthritis and hyperuricemia renal fibrosis. Therefore, the relaxin signaling pathway may be an effective way to treat gout and hyperuricemia nephropathy.

### 4.5. TNF Signaling Pathway

**Figure 10:**
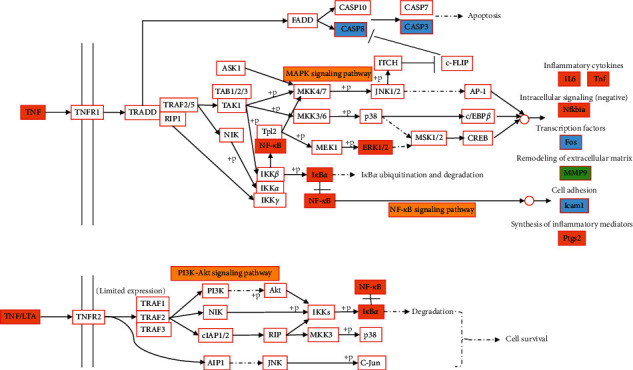
TNF signaling pathway in the treatment of gout and hyperuricemia with plantain. Note: orange is the common target of gout and hyperuricemia, green is the target of gout, blue is the target of hyperuricemia, and yellow is the other signaling pathways that may be involved in this pathway.

TNF-*α* is a proinflammatory cytokine, which can induce RA to secrete lipopolysaccharide to promote the proliferation of fibroblast-like synovial cells, promote the expression of inflammatory factors such as IL-1*β* and IL-6, and increase the phosphorylation of ERK [67]. TNF-*α* was significantly increased in MSU crystals-induced gouty joints of mice and was positively correlated with the severity of arthritis [68]. Clinical studies have shown that the TNF-*α* inhibitor etanercept can significantly improve the clinical manifestations and laboratory results of gouty arthritis [69].  Figure 10 shows the results of TNF signaling pathway analysis. In the hyperuricemia rat model, serum UA, TNF-*α*, IL-6, and lipopolysaccharide were increased, indicating that hyperuricemia is in a mildly systemic inflammatory state [70]. Previous studies have shown that plantain has significant anti-inflammatory activity and can reduce the secretion of TNF-*α*, IL-10, and IL-6 in the mouse leukemia cells of monocyte macrophage (RAW264.7) inflammatory model [71, 72]. It has been proved that compounds dinatin [73], baicalein [35], baicalin [35], sitosterol [74], stigmasterol [44], and luteolin [45] can reduce the level of TNF-*α*. This suggests that plantain may be a potential treatment for gout and hyperuricemia by inhibiting TNF signaling pathway.

### 4.6. AGE-RAGE Signaling Pathway in Diabetic Complications

**Figure 11:**
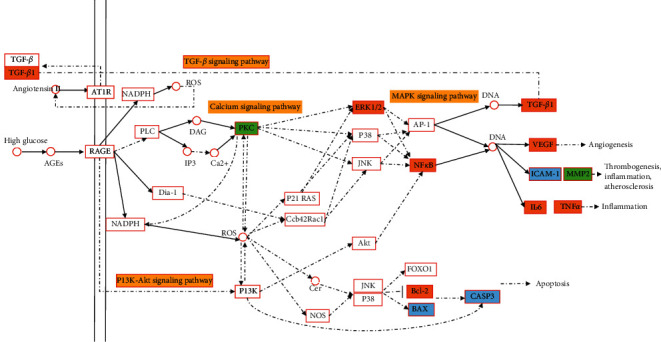
AGE-RAGE signaling pathway in diabetic complications in the treatment of gout and hyperuricemia with plantain. Note: orange is the common target of gout and hyperuricemia, green is the target of gout, blue is the target of hyperuricemia, and yellow is the other signaling pathways that may be involved in this pathway.

Advanced glycosylation end products (AGEs) are the products of nonenzymatic synthesis of reducing sugars combined with lipids, proteins, and nucleic acids. AGEs combine with AGEs receptors receptor for advanced glycation end products (RAGE) to induce oxidative stress and inflammation in various types of cells and organs [75, 76].  Figure 11 shows the analysis results of AGE-RAGE signaling pathway in diabetic complications. Studies have shown that AGEs combined with RAGE can significantly increase the expression levels of TGF-*β* and MMP-9 mRNA, IL-1, and protein in OA chondrocytes, significantly reduce the activity of catalase and superoxide dismutase (SOD), increase the level of malondialdehyde, and significantly promote the nuclear translocation of NF-*κ*B [77]. The expression levels of TGF-*β* and MMPs can be used as experimental observation indicators to judge the severity of OA [77]. The study found that when UA stimulated human umbilical vein endothelial cells, the mRNA expression of RAGE and high-mobility group box 1 protein (HMGB1) increased, and HMGB1 combined with RAGE activated NF-*κ*B signaling, promoting the release of inflammatory cytokines IL-6 and TNF-*α* and adhesion molecules intercellular cell adhesion molecule-1 (ICAM-1), and vascular cell adhesion molecule-1 (VCAM-1) [78]. There was a positive association between serum RAGE levels and serum UA in patients with hyperuricemia [79]. Therefore, RAGE is critical in the development of hyperuricemia and gout diseases. Experiments showed that, in the human keratinocyte cell line (HaCaT) and primary human dermal fibroblasts (HDF) cell experiments, plantain could reduce the expression of MMP-1 and proinflammatory cytokines induced by ultraviolet radiation B (UVB) and AGEs by inhibiting the phosphorylation of MAPKs, reduce the NF-*κ*B nuclear translocation by inhibiting the I*κ*B*α* phosphorylation, and reduce upregulation of RAGE [80]. In summary, the AGE-RAGE signaling pathway in diabetic complications may be an effective way for plantain to treat gouty arthritis.

### 4.7. Correlation Analysis of Active Compounds with Gout and Hyperuricemia

It has been proved that plantain has significant effects such as reducing UA level [40], reducing XOD activity [81], anti-inflammatory and analgesic [12], and protecting kidney [15], so it is a common drug in clinical treatment of gout. Studies have shown flavonoids have strong anti-inflammatory activity [82]. The correlation analysis of 7 active compounds with gout and hyperuricemia diseases is as follows.

Dinatin, also known as hispidulin, can treat mast cell-mediated allergic inflammation by inhibiting JNK phosphorylation and downregulating the expression of TNF-*α* and IL-4 inflammatory factors [73]. It can inhibit the production of proinflammatory cytokines such as TNF-*α*, IL-1*β*, and IL-6 induced by alginic acid [83]. Dinatin can inhibit the expression of HIF-1*α* protein by activating AMPK pathway [52]. It can inhibit the production of inducible nitric oxide synthase (iNOS) protein and inflammatory cytokines such as TNF-*α*, IL-1, and IL-8 induced by lipopolysaccharide in Raw264.7 and human colon cancer (HT29) cells [84]. It can also be used to treat osteoporosis and prevent bone loss by activating AMPK signaling [85]. Xanthine oxidase (XOD) is a key enzyme in purine catabolism, which catalyzes hypoxanthine and xanthine to UA in human metabolism. Therefore, inhibition of XOD activity is the main clinical method to treat hyperuricemia and gout [86]. In vitro experiments have shown that hispidulin has a significant inhibitory effect on XOD [87]. Therefore, dinatin may play an anti-inflammatory effect through TNF and HIF-1*α* pathways. And based on its antixanthine oxidase activity, it may be a potential compound for the treatment of gout and hyperuricemia.

Baicalein has obvious antioxidant and anti-inflammatory activities, which can improve various kinds of inflammation, such as gout and RA, cardiovascular disease, respiratory disease, and inflammatory bowel disease [35]. Studies have shown that baicalein plays an antifibrosis role in the treatment of respiratory diseases by inhibiting TGF-*β*/Smad signaling pathway [35]. The production of IL-6, IL-8, and MCP-1 in human essential cells (HMCS) induced by IL-1*β* and TNF-*α* was also inhibited by inhibiting the activation of NF-*κ*B and the phosphorylation of I*κ*B*α* [35]. Baicalein also inhibits the accumulation and activation of HIF-1*α* protein and the expression of iNOS by inhibiting the activation of phosphatidylinositol 3-kinase/protein kinase B (PI3K/Akt) and the production of reactive oxygen species (ROS) in the BV2 microglia [88]. Baicalein can also prevent retinal ischemia by reducing the levels of HIF-1*α*, VEGF, and MMP-9 [53]. It can also reduce the expression of IL-17 protein to treat autoimmune uveoretinitis [42]. Baicalein can inhibit the production of nitric oxide and the activation of caspase-3/8 induced by IL-1*β* and TNF-*α* in OA chondrocytes and reduce the production of MMP-1, MMP-3, and MMP-13 [89, 90]. It can also reduce the activity of serum UA and XOD to treat hyperuricemia, prevent renal fibrosis by inhibiting MMP-7 and MMP-9 signals, and improve renal damage caused by hyperuricemia [91]. Therefore, baicalein may regulate TNF, HIF-1, and IL-17 pathways to treat gout and hyperuricemia.

Baicalin can achieve antipulmonary fibrosis effect by reducing the levels of TGF-*β*1 and pERK1/2 [35]. Baicalin can reduce lung cancer metastasis by reducing HIF-1*α* level [54] and can reduce the expression levels of RAGE, IL-6, and TGF-*β*1 to improve pulmonary hypertension [92]. It can reduce the expression of IL-6, TNF, CXCL1, CXCL10 (chemokine (C-X-C motif) ligand 10), and MMP3 genes in mice to treat OA [93]. It can also inhibit the Janus kinase 1/signal transducer and activator of transcription 3 (JAK1/STAT3) signaling pathway to reduce the expression of TNF-*α*, IL-1*β*, IL-6, MMP-2, MMP-9, iNOS, and COX-2 in RA [94]. In the adjuvant-induced arthritis mice models, baicalin can control inflammation by blocking the IL-17 pathway and reducing the expression of IL-6, TNF-*α*, ICAM-1, and VCAM-1 [35]. It also showed significant inhibition of XOD [95]. Therefore, baicalin may treat gout and hyperuricemia by regulating IL-17, TNF, and HIF-1 pathways.

Sitosterol can treat cerebral aneurysms by reducing the expression of chemokines and inflammatory cytokines TNF-*α*, IL-8, IL-1*β*, IL-17, IL-6, and MMP-2/9 [43]. It can reduce the level of IL-17, TNF-*α*, IL-1*β*, IL-6, and IL-12 in RA mice and promote the production of anti-inflammatory cytokine IL-10 [44, 74]. Sitosterol can also treat foot edema caused by MSU crystals in mice [96]. Therefore, sitosterol may play an antigout role by regulating IL-17 and TNF pathways.

6-OH-luteolin is also known as 6-hydroxyluteolin. Previous studies have shown that 6-hydroxyluteolin can reduce serum UA levels by inhibiting liver XOD and xanthine dehydrogenase (XDH) activity to treat hyperuricemia diseases [97]. At present, it has not been studied whether this compound has anti-inflammatory activity. The mechanism of 6-OH-luteolin in the treatment of gout and hyperuricemia needs to be studied.

Stigmasterol can decrease the levels of IL-17, TNF-*α*, and IL-1*β* in rheumatoid arthritis [44], can significantly reduce the plantar edema induced by MSU crystals, and can reduce the serum UA levels in hyperuricemia mice by inhibiting liver XOD activity [98, 99]. It is a potential active compound for the treatment of gout and hyperuricemia.

Luteolin has high anti-inflammatory activity and can effectively inhibit the expression of NLRP3 by inhibiting IL-17A signal in enteritis tissue [100]. Luteolin can inhibit the activation of HIF-1 and STAT3 signal and reduce the expression of VEGF and MMP-9 to play anticancer role [55]. It can inhibit the levels of TNF-*α*, IL-6, IL-1*β*, IL-17, membrane cation-selective receptor channels (P2X4), nucleotide oligomerization domain-like receptor protein 1 (NLRP1), apoptosis-associated speck-like protein containing CARD (ASC), and caspase-1p10 in RA rats induced by Freund's complete adjuvant and reduce the infiltration of inflammatory cells and synovial hyperplasia [45]. It can reduce the levels of IL-1*β* and TNF-*α* to treat paw swelling and inflammation caused by MSU crystal [101]. It can also reduce the level of urate transporter 1 (mURAT1), inhibit the activity of XOD and increase the excretion of UA to treat hyperuricemia, and can prevent renal insufficiency caused by gout and hyperuricemia in the late stage [101]. Therefore, the compound luteolin may treat gout and hyperuricemia through TNF, IL-17, and HIF-1 pathways.

## 5. Conclusion

In summary, this study based on network pharmacological analysis and experimental verification revealed that plantain may be used to treat gout and hyperuricemia through controlling inflammatory factors and immunomodulation. It has the characteristics of multicomponent, multitarget, and multichannel. It provides better guidance for subsequent experiments and research and also provides new ideas and new approaches for the mechanism and drug development of plantain for the treatment of gout and hyperuricemia.

## Figures and Tables

**Table 1:** Potential active compounds of plantain.

Mol ID	Molecule name	OB (%)	DL
MOL001735	Dinatin	30.97	0.27
MOL002714	Baicalein	33.52	0.21
MOL002776	Baicalin	40.12	0.75
MOL000359	Sitosterol	36.91	0.75
MOL004004	6-OH-luteolin	46.93	0.28
MOL000449	Stigmasterol	43.83	0.76
MOL000006	Luteolin	36.16	0.25
MOL007783	Melampyroside	57.50	0.80
MOL007796	Stigmasteryl palmitate	38.09	0.40
MOL007799	*β*-Sitosteryl palmitate	30.91	0.40

**Table 2:** Information table of active compounds of plantain.

CAS number	Compound name	Chemical structure	Molecular formula	Molecular weight
1447-88-7	Dinatin	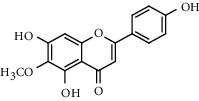	C_16_H_12_O_6_	300.27
491-67-8	Baicalein	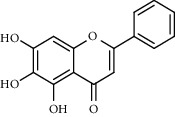	C_15_H_10_O_5_	270.24
21967-41-9	Baicalin	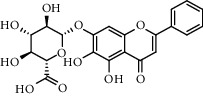	C_21 _H_18 _O_11_	446.36
83-46-5	Sitosterol	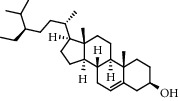	C_29 _H_50 _O	414.71
18003-33-3	6-OH-luteolin	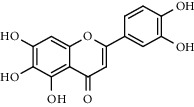	C_15_H_10_O_7_	302.24
83-48-7	Stigmasterol	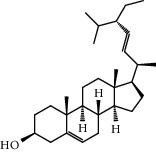	C_29_H_48_O	412.70
491-70-3	Luteolin	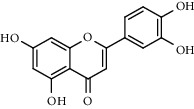	C_15_H_10_O_6_	286.24

## Abbreviations

TCMSP: Traditional Chinese medicine systems pharmacology database and analysis platform
TTD: Therapeutic Target Database
OMIM: Online Mendelian Inheritance in Man
GO: Gene Ontology
KEGG: Kyoto Encyclopedia of Genes and Genomes
TNF-*α*: Tumor necrosis factor-*α*
HIF-1: Hypoxia-inducible factor-1
IL: Interleukin
UA: Uric acid
MSU: Monosodium urate
XOD: Xanthine oxidase
TCM: Traditional Chinese medicine
OB: Bioavailability
DL: Drug-likeness
ADME: Absorption, distribution, metabolism, and excretion
PPI: Protein-protein interaction
MIPS: Munich Information Center for Protein Sequences
RA: Rheumatoid arthritis
OA: Osteoarthritis
MMP: Matrix metalloproteinase
COX-2: Cyclooxygenase-2
CCL5: Chemokine C-C motif ligand 5
NF-*κ*B: Nuclear factor-kappa B
NLRP3: Nucleotide binding oligomerization domain-like receptor protein 3
AGE-RAGE: Advanced glycation end products-receptor for advanced glycation end products
CXCL1/KC: Chemokine (C-X-C motif) ligand 1/keratinocyte-derived chemokine
AP-1: Activator protein 1
I*κ*B*α*: Inhibitor of NF-*κ*B
JNK: Jun N-terminal kinases
ERK: Extracellular signal-regulated kinase
VEGF: Vascular endothelial growth factor
MCP-1: Monocyte chemotactic protein 1
NGF: Nerve growth factor
HGF: Hepatocyte growth factor
Akt: Protein kinase B
CCL2: Chemokine C-C motif ligand 2
RXFP1: Relaxin/insulin-like peptide receptor 1
TGF-*β*1: Transforming growth factor-*β*1
RAW264.7: Mouse leukemia cells of monocyte macrophage
RAGE: Receptor for advanced glycation end products
SOD: Superoxide dismutase
HMGB1: High-mobility group box 1 protein
ICAM-1: Intercellular cell adhesion molecule-1
VCAM-1: Vascular cell adhesion molecule-1
HaCaT: Human keratinocyte cell line
HDF: Primary human dermal fibroblasts
UVB: Ultraviolet radiation B
iNOS: Inducible nitric oxide synthase
HT29: Human colon cancer cells
HMCS: Human essential cells
PI3K/Akt: Phosphatidylinositol 3-kinase/protein kinase B
ROS: Reactive oxygen species
CXCL10: Chemokine (C-X-C motif) ligand 10
JAK1/STAT3: Janus kinase 1/signal transducer and activator of transcription 3
XDH: Xanthine dehydrogenase
P2X4: Membrane cation-selective receptor channels
NLRP1: Nucleotide oligomerization domain-like receptor protein 1
ASC: Apoptosis-associated speck-like protein containing CARD
URAT1: Urate transporter 1.
